# Increased Time to Provider for Patients With a Non-English Language Preference: A Retrospective Cohort Study

**DOI:** 10.1016/j.acepjo.2025.100239

**Published:** 2025-08-29

**Authors:** Asmaa Rimawi, Anne Sung, Morgan Pike, Erica Lin, David A. Haidar, Chiu-Mei Chen, Richard Medlin, Marcia Perry, Christopher M. Fung

**Affiliations:** Department of Emergency Medicine, University of Michigan, Ann Arbor, Michigan, USA

**Keywords:** language preference, non-English language preference, patients, emergency department crowding, health disparities

## Abstract

**Objectives:**

We assessed time to provider (TTP) for patients with a non-English language preference (NELP) compared to patients with an English language preference (ELP) in the emergency department (ED).

**Methods:**

We conducted a retrospective cohort study of adults presenting between 2019 and 2023 to a large urban ED. We used a 2-step classification that first identified NELP from patients’ reported language at registration, followed by identification in the narrative text of the triage note. We compared TTP among patients with a triage note-identified NELP, triage note-unidentified NELP, and ELP, including at night and during times of ED crowding.

**Results:**

Among 262,203 visits, 8227 were patients with a NELP, of which 3375 were triage note-identified NELP. The median TTP was 11 minutes for patients with an ELP (95% CI, 11-11), 15 minutes for triage note-identified NELP (14-16), and 13 minutes for triage note-unidentified NELP (12-14). During times of ED crowding, patients with an ELP had a median TTP of 12 minutes (95% CI, 12-12) compared with 21 minutes for patients with a triage note-identified NELP (17-27) and 17 minutes for those with a triage note-unidentified NELP (14-20). At nighttime, the median TTP was 19 minutes for patients with an ELP (95% CI, 19-19) compared with 28 minutes for triage note-identified NELP (25-32) and 25 minutes for triage note-unidentified NELP (22-28).

**Conclusion:**

Patients with a NELP experience longer TTP, especially those whose triage note identifies their language status. ED crowding and nighttime are associated with further increases in TTP among patients with a NELP.

**The Bottom Line**

This retrospective analysis aimed to evaluate the differences in the time it took for patients to be picked up by a provider at an urban, academic emergency department (ED). Patients who had a documented a non-English language preference experienced longer wait times before a provider assigned themselves compared with those with an English language preference. These wait time disparities further increased during periods of high waiting room (WR) volume or decreased provider availability. As boarding and WR volumes continue to rise in EDs nationwide, the unique impacts on patients with language barriers must be carefully considered.

## 1 Introduction

### 1.1 Background

Between 2010 and 2019, the percentage of the US population who prefer to speak a language other than English rose from 9% to 22%, according to the US Census Bureau. Patients with a non-English language preference (NELP) report less satisfaction in their emergency department (ED) care,^1^, ^2^, ^3^, ^4^ experience increased rates of unplanned ED revisits,^5^^,^^6^ and have suboptimal disease outcomes compared with patients with an English language preference (ELP).^7^^,^^8^ As the number of Americans with a NELP continues to rise, understanding the impact of a NELP on care received in the ED is critical. Simultaneously, prolonged waiting times in the ED have become more common given increased patient volumes and increased boarding.^9^^,^^10^ Prolonged wait times have been associated with higher percentages of patients leaving without being seen,^11^ decreased overall satisfaction,^12^ and overall poor outcomes.^13^

### 1.2 Importance

The US Civil Rights Act of 1964 dictates that hospitals must provide language and interpreter assistance, with the Department of Health and Human Services expanding on these requirements in 1980 by dictating against discrimination of patients with a NELP.^14^ Despite these stipulations, the experiences of patients with a NELP in the ED regarding performance and quality of care metrics continue to be unclear. Although previous studies have indicated that patients with a NELP are undertriaged^15^ and experience queue jumps when waiting for provider care,^16^ there are no studies, to our knowledge, that have directly assessed wait times for patients with a NELP in the ED. With increased ED boarding and known deleterious outcomes associated with prolonged wait times, understanding the impact of NELP on wait times is critical for identifying barriers to care in the ED for patients with a NELP, guiding resources, and improving disparities in care.^17^^,^^18^ Additionally, best practice guidelines on the care of patients with a NELP do not discuss methods to minimize disparities regarding care metrics such as wait time.^19^

### 1.3 Goals of This Investigation

We examined provider wait times as a first step in creating a systematic assessment of barriers to the highest standard of care for patients with a NELP. This study explores time to provider (TTP) for patients with a NELP compared with patients with an English language preference (ELP). We hypothesized that TTP is longer for patients with a NELP compared with patients with an ELP, and, that, TTP is longer for patients with a NELP when NELP is identified within a patient’s triage documentation.

## 2 Methods

### 2.1 Study Design and Setting

We conducted a retrospective cohort study of patients aged >21 years who presented to the ED between July 1, 2019, and June 30, 2023, at a single, large urban academic medical center in Michigan with ∼80,000 adult visits per year. All data were collected from the electronic health record (Epic Systems). This study was approved by the institutional review board and research ethics committee at the University of Michigan (HUM00185610). The institutional review board waived the requirement for informed consent.

In our ED, patients are seen by residents (both emergency medicine and off-service rotators) or physician assistants supervised by attending emergency physicians in addition to patients seen primarily by attending physicians only. Patients with an Emergency Severity Index (ESI) of 1 are assigned providers and seen immediately in the resuscitation bays. Patients with an ESI of 2 to 5 are roomed and await a provider to assign themselves to the patient. Providers are encouraged to see new patients in order of ESI priority followed by length of stay but may exercise their own discretion. Patients with ESI of 5 are seen either in triage prior to rooming or as nurse-only visits. When interpreter services are required, a nurse typically makes the request to facilitate communication during the triage process. Providers typically request interpreter services after they have assigned themselves to a patient or during their evaluation. Interpreter services via telephone are always available for any language. In-person interpreter services for some languages are also available but do not provide 24/7 coverage. A detailed description of our ED workflow is presented in Figure S1.

### 2.2 Selection of Participants

**Figure 1:**
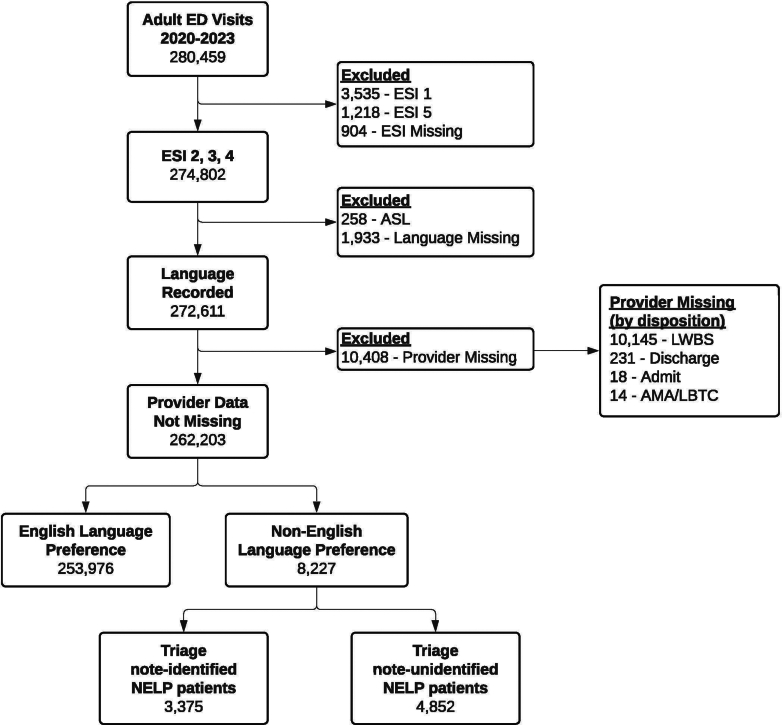
Study enrollment diagram. All adult visits to the ED (age > 21 years) were initially included, and after exclusions for ESI 1, 5, and missing acuity level; ASL, missing language data; and missing provider data, a total of 262,203 visits were included. Visits with missing provider data were further characterized by the recorded disposition. 10,145/10,408 (97.5%) of the visits missing provider data were LWBS. AMA, against medical advice; ASL, American sign language; ED, emergency department; ESI, Emergency Severity Index; LBTC, left before treatment complete; LWBS, left without being seen.

Patients aged >21 years who presented to the ED between July 1, 2019, and June 30, 2023, were considered for analysis. Visits with an ESI of 1 or 5, missing ESI, missing language preference, or an American sign language preference were excluded. Patients with an ESI of 1 or 5 were excluded as these patients are seen within special treatment areas in our department. Additionally, American sign language patients use a different pathway for interpreter services in triage and were thus excluded. Patients who presented to the ED but were dispositioned before a provider was assigned to their care, which included those who left from the waiting room (WR) without being roomed, those who were admitted or discharged from the WR by a triage physician, or those who left without being seen or before completion of treatment, were not included in this analysis. Counts of excluded patient encounters at each level are detailed in the study enrollment diagram (Fig 1).

### 2.3 Exposure Variables

A 2-step classification method was used to identify the primary exposure variable, language preference status. First, patients were classified as NELP or ELP based on language preference reported at registration. All patients who presented to the ED were asked about their language preference by registration staff as part of the intake process upon their arrival to the ED. After initial classification of patients as NELP or ELP based on their language preference reported at registration, patients with a NELP were further classified based on identification of their NELP within the text of their triage note.

The triage note of any patient categorized as having a NELP at registration was exported into a web-based data capture system and reviewed by the study team. Patients with triage notes that identify their NELP status were classified as patients with a triage note-identified NELP, while patients with triage notes that do not identify their NELP status were classified as patients with a triage note-unidentified NELP. To perform this classification, the study team reviewed 100 randomly selected triage notes from patients with a NELP and created criteria for the classification of triage note-identified patients with a NELP. Criteria for classification as patients with a triage note-identified NELP were (i) explicit statement of a non-English preference, (ii) mention of need for an interpreter, (iii) mention of a visitor serving as an interpreter, (iv) mention that a family member is available via phone for interpretation, or (v) mention that a hospital or phone interpreter was used to obtain triage information. All triage notes from patients with a NELP were then reviewed by study team members (resident or attending emergency physicians) to classify all visits for patients with a NELP as triage-note identified and unidentified. The electronic form used to collect these data is reproduced in Figure S2.

### 2.4 Outcomes

The primary outcome of the study was TTP, which was defined as time in minutes from when a patient was moved to a treatment area within the ED (including hallway or vertical treatment beds) to the time a provider assigned themselves to the patient (Fig S1). In this context, provider refers to the resident, physician assistant, or attending physician assigned to a patient in the emergency department. Movement to a treatment area and provider assignment time are recorded as time stamps, and these data were extracted from the electronic health record. TTP does not include the time required to obtain a phone or in-person interpreter as this generally occurs after provider assignment (Fig S1).

### 2.5 Data Collection and Other Measurements

All other variables including race, ethnicity, age (years), sex, WR and boarding patients, ESI, time and mode of arrival, insurance status, and disposition were extracted from the electronic health record. These data are used for the clinical operations of our department, and the database is maintained by a department analytics team. An electronic query to this database was used to collect all electronic health record-derived data for this study except for the triage note-based NELP identification.

### 2.6 Analysis

The goal of our study was to determine if patients with a NELP have a longer TTP. Median TTP was estimated using the Kaplan-Meier method and a 3-level comparison between patients with an ELP and patients with a NELP with and without a triage note identifier of their NELP status. A multivariable accelerated failure time (AFT) model was used to estimate the independent association between patients’ NELP status and TTP.^20^ The AFT framework was selected because the goal of this study was to directly model a change in time to event and because the data did not meet the proportional hazards assumption. Our model included the following covariates: ESI acuity level, time of day of patient arrival, and number of boarding and WR patients at patient arrival. These covariates were selected to capture the effects of patient acuity (ESI), staffing (day vs night), crowding (WR and boarding patients), and are known to influence provider wait times in our ED. The number of WR and boarding patients were binned rather than treated as a continuous variable for ease of interpretation and to capture nonlinear relationships while preserving ordinality. The log-normal error distribution, which does not require proportional hazards, was determined to be the best fit graphically using residual plots and Akaike information criterion. No significant interactions were detected also based on fit statistics. Homoscedasticity was acceptable and determined graphically using residual plots. Model coefficients were exponentially transformed and reported as time ratios (TRs). To explore the impact of ED demand on TTP, 2 prespecified subanalyses were completed. For our institution, we hypothesized that the greatest times of stress on the ED were when the WR had >30 patients or at nighttime, defined as 7 pm to 7 am arrival, when the ED was staffed with fewer medical providers. Thus, we completed 2 subgroup analyses: TTP during times of ED crowding (defined as >30 patients in the WR) and at nighttime (measured by arrival window between 7 pm and 7 am). There was no missing data for variables used in multivariable modeling. All statistical analyses and final data preparation were completed in RStudio (version 2023.12.1, Posit Software) using R version 4.3.3 (R Foundation).

## 3 Results

### 3.1 Characteristics of Study Subjects

During the study (from July 1, 2019, to June 30, 2023), there were 280,459 visits by adults aged >21 years to our ED. After exclusions for missing data and ESI 1 and 5 visits, 262,203 visits (93%) were included for analysis (Fig 1). Most visits excluded for missing provider data were because of patients who left without being seen (10,145/10,408, 97.5%). The rate of left without being seen in these excluded patients was 97.5% (n = 9820) for patients with an ELP and 97.9% (n = 325) for patients with a NELP. In the final cohort of included visits, 253,976 (96.9%) were patients with an ELP, and 8227 (3.1%) visits were patients with a NELP. Among patients with a NELP documented at the time of registration, 3375 (41%) had a triage note indicating their NELP status. Triage note-identified patients with a NELP were more likely to be female and older compared with ELP and triage note-unidentified patients with a NELP. Triage note-unidentified patients with a NELP were lower acuity with more patients triaged as ESI 4 and fewer as ESI 2. The proportion of visits by language preference and ESI were stable during the study period (Fig S3). The number of WRs and boarding patients, the type of medical provider, and the time of arrival after 7 pm were not significantly different between ELP and either of the NELP groups. Patient encounter characteristics are summarized in Table 1, and the tabulation of NELP patients by preferred language is presented in Table S1.

**Table 1:** Demographics of patients included in the final analysis by language preference.

Characteristic	English language preference n (%)	Triage-identified patients with NELP n (%)	Triage note-unidentified patients with NELP n (%)	SMD
Sex				0.107
Female	135,099 (53.2)	2062 (61.1)	2655 (54.7)	
Male	118,868 (46.8)	1313 (38.9)	2197 (45.3)	
Unknown	9 (0.0)	0 (0.0)	0 (0.0)	
Race				1.054
AIAN	1550 (0.6)	16 (0.5)	9 (0.2)	
Asian	7305 (2.9)	791 (23.4)	1224 (25.2)	
Black or African American	45824 (18.0)	103 (3.1)	224 (4.6)	
Choose not to disclose	1072 (0.4)	17 (0.5)	15 (0.3)	
MENA	476 (0.2)	76 (2.3)	169 (3.5)	
Missing or unknown	1262 (0.5)	47 (1.4)	54 (1.1)	
NHPI	307 (0.1)	2 (0.1)	5 (0.1)	
Other	8008 (3.2)	1240 (36.7)	1620 (33.4)	
White or Caucasian	188,172 (74.1)	1083 (32.1)	1532 (31.6)	
Waiting room volume				0.063
0-9	132,826 (52.3)	1749 (51.8)	2429 (50.1)	
10-19	54,341 (21.4)	693 (20.5)	1025 (21.1)	
20-29	38,006 (15.0)	543 (16.1)	760 (15.7)	
30-39	21,013 (8.3)	272 (8.1)	475 (9.8)	
40-49	6791 (2.7)	98 (2.9)	147 (3.0)	
50+	999 (0.4)	20 (0.6)	16 (0.3)	
Emergency Severity Index				0.149
2	118,117 (46.5)	1423 (42.2)	1822 (37.6)	
3	114,274 (45.0)	1685 (49.9)	2441 (50.3)	
4	21,585 (8.5)	267 (7.9)	589 (12.1)	
7 pm-7 am arrival	82,118 (32.3)	1005 (29.8)	1583 (32.6)	0.041
Disposition				0.112
Admit	106,326 (41.9)	1352 (40.1)	1685 (34.7)	
AMA or LBTC	2749 (1.1)	33 (1.0)	42 (0.9)	
Deceased	128 (0.1)	0 (0.0)	2 (0.0)	
Discharge	143,451 (56.5)	1977 (58.6)	3082 (63.5)	
Left without being seen	1322 (0.5)	13 (0.4)	41 (0.8)	
Insurance				0.226
Medicare or Medicaid	51,089 (20.1)	1047 (31.0)	1098 (22.6)	
None listed	46,004 (18.1)	771 (22.8)	1109 (22.9)	
Private	156,883 (61.8)	1557 (46.1)	2645 (54.5)	
Medical provider				0.063
Attending only	21,054 (8.3)	219 (6.5)	419 (8.6)	
House officer	121,137 (47.7)	1570 (46.5)	2258 (46.5)	
Physician assistant	111,785 (44.0)	1586 (47.0)	2175 (44.8)	
Age (y) (mean SD)	52.43 (19.08)	58.90 (20.08)	52.84 (19.65)	0.219

AIAN, American Indian or Alaska Native; AMA, against medicine advice; LBTC, left before treatment complete; MENA, Middle Eastern of North African; NELP, non-English language preference; NHPI, Native Hawaiian and Other Pacific Islander; SMD, standardized mean difference.

### 3.2 Main Results

**Figure 2:**
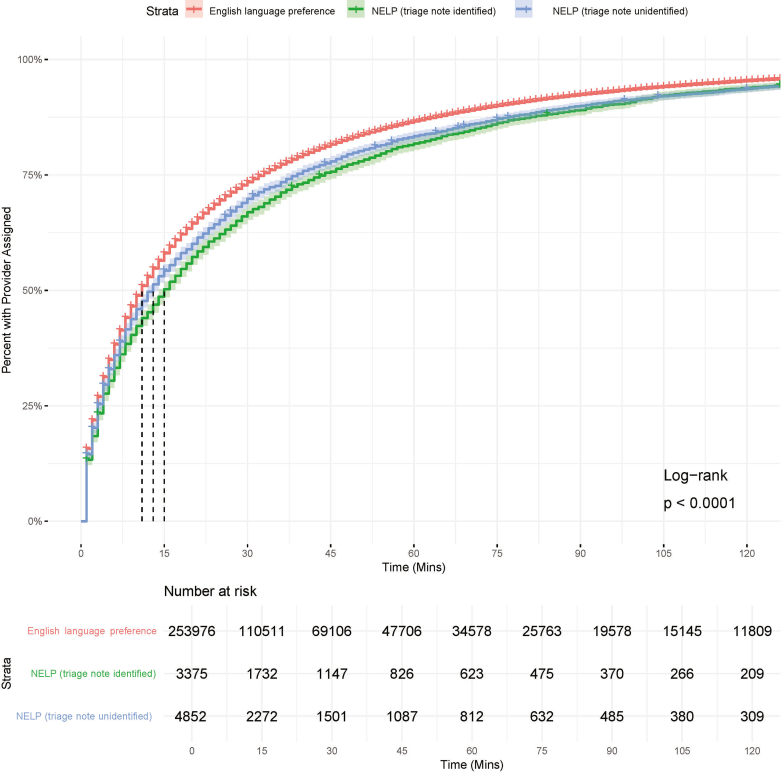
Kaplan-Meier survival curve time to provider by NELP status. NELP status is further subdivided to NELP triage-note identified and unidentified in which “triage note identified” indicates that the patients NELP status is identified in the text of the brief note as documented by a triage nurse. NELP, non-English language preference.

The median TTP was 11 minutes for patients with an ELP (95% CI, 11-11), 15 minutes for triage-identified patients with a NELP (95% CI, 14-16), and 13 minutes for triage note-unidentified patients with a NELP (95% CI, 12-14). The unadjusted Kaplan-Meier curves for each of these groups are shown in Figure 2. The median TTP for the full cohort and subgroups is summarized in Table 2. The median TTP was greater in triage-identified patients with a NELP compared with triage note-unidentified patients with an ELP and a NELP across ESI 2, 3, and 4 visits. When compared to patients with an ELP, triage-identified patients with a NELP waited a median of 3, 6, and 10 minutes longer during ESI 2, 3, and 4 visits, respectively.

**Table 2:** Median time to provider for patients with an ELP, triage-identified patients with a NELP, and triage note-unidentified NELP by ESI level, WR volume, and arrival time.

	n	Median	95% CI
Full cohort			
ELP	253,976	11	11-11
Triage-identified NELP	3375	15	14-16
Triage note-unidentified NELP	4852	13	12-14
ESI level 2			
ELP	118,117	9	9-9
Triage-identified NELP	1423	12	10-13
Triage note-unidentified NELP	1822	9	9-10
ESI level 3			
ELP	114,274	14	14-15
Triage-identified NELP	1685	20	18-22
Triage note-unidentified NELP	2441	17	15-19
ESI level 4			
ELP	21,585	15	14-15
Triage-identified NELP	267	25	16-33
Triage note-unidentified NELP	589	14	11-19
WR > 30			
ELP	66,809	12	12-12
Triage-identified NELP	933	21	17-27
Triage note-unidentified NELP	1398	17	14-20
7 pm-7 am arrival			
ELP	82,118	19	19-19
Triage-identified NELP	1005	28	25-32
Triage note-unidentified NELP	1583	25	22-28

ELP, English language preference; ESI, Emergency Severity Index; NELP, non-English language preference; WR, waiting room.

During times of ED crowding (WR > 30), patients with an ELP had a median TTP of 12 minutes (95% CI, 12-12) compared with 21 minutes for triage-identified patients with a NELP (95% CI, 17-27) and 17 minutes for triage note-unidentified patients with a NELP (95% CI, 14-20). In the subgroup of patients who presented between 7 pm and 7 am, the median TTP was 19 minutes for patients with an ELP (95% CI, 19-19) compared with 28 minutes for triage-identified patients with a NELP (95% CI, 25-32) and 25 for triage note-unidentified patients with a NELP (95% CI, 22-28).

**Figure 3:**
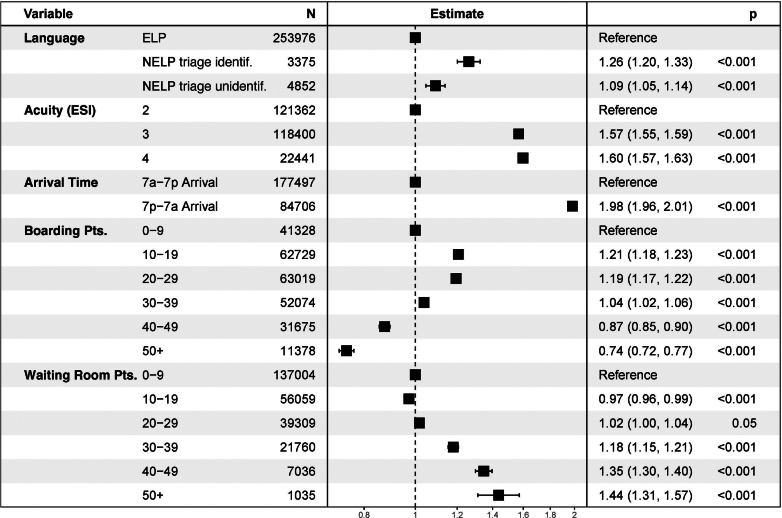
Forest plot for a log-normal AFT model. An AFT model adjusting for the effects of patient acuity, arrival time, and the number of boarding or waiting room patients at arrival was used to estimate the association between NELP status and TTP. Point estimate and 95% confidence intervals reported are expressed in time ratios (ratio of exponentiated model coefficients). NELP status with identification of this status in the triage note imparts a 26% increase in TTP. AFT, accelerated failure time; NELP, non-English language preference; TTP, time to provider.

We used an AFT model to adjust for the effects of acuity, arrival time, boarding, and WR patients at arrival on TTP. In this multivariable model (Fig 3), triage-identified status among patients with a NELP was associated with a 26% delay in TTP (TR, 1.26 [95% CI, 1.20-1.33]), and triage note-unidentified status among patients with a NELP was associated with a 9% delay in TTP (TR, 1.09 [95% CI, 1.05-1.14]) compared with the status among patients with an ELP.

## 4 Limitations

Although this study demonstrates increased TTP for patients with a NELP compared with patients with an ELP, it has several limitations. First, this study was conducted at a single urban academic medical center and may not apply to other hospital systems. In addition, only 3.1% of patients in our study had a NELP. As such, our findings may not extend to other centers with higher percentages of patients with a NELP, where access to resources for interpretation such as readily available in-person interpreters may be more streamlined. Future multicenter studies with a higher proportion of patients with a NELP may be required to replicate our findings. Additionally, although previous studies have indicated bias associated with ESI assignment, specifically undertriage of patients with a NELP,^15^ any bias associated with ESI assignment was not accounted for within this study. If undertriage of patients with a NELP were to occur in our cohort, the delay in TTP could be increased.

Race was not included within our multivariable analysis. For providers to identify a patient’s race prior to assigning themselves to the patient, they would be required to enter the patient’s chart and search for the patient’s race. Given that race is not easily accessible to a provider when they are assigning themselves to patients, we did not adjust for race in this analysis. However, previous studies have suggested an association between race and wait times.^21^ Unlike race, age (years) and sex are available to the provider before they assign themselves to a patient and may impart a biasing effect on TTP that was not included in our multivariable analysis.

Missing data leading to exclusions from the cohort may have caused a sample bias to occur. Missing language preference can occur if a patient is discharged before the registration process has been completed, if the patient declines to answer, or if the patient is unable to answer. Missing ESI is likely to occur in urgent situations in which patients are escorted to critical rooms or operating rooms with minimal ED interaction. Should they have been given an ESI, it would likely have been 1, and they would have thus been excluded from the study. Given that missing data accounted for <1.1% of the data, potential bias on the outcome is likely to be minimal. Patient encounters excluded by missing provider assignments accounted for 3.7% of the data although the vast majority (97.5%) of these exclusions were because the patient left prior to being seen.

Our analysis did not account for the ability of the primary provider or supervising provider (if seen by a physician assistant or house officer) to communicate in a patient’s preferred language or the effect of third parties such as a family member or other staff serving as an ad hoc interpreter. We did not collect data on languages spoken by medical providers, nursing, or other staff. Finally, NELP status represents a spectrum of true English proficiency and some patients who indicated a NELP may, in fact, have been able to communicate effectively in English, but this was not studied. Thus, these confounders may influence TTP, but the net effect remains unmeasured.

## 5 Discussion

Patients with a NELP often experience disparities during their encounters with the health system. In the ED, interpreter services cannot be prearranged, and the necessary, recurrent points of contact between patient and provider (history and physical examination, explanation of multiple test results as they result, reassessments, discharge instructions, etc) may lead to delays during all stages of care.

Our analysis demonstrates an association between TTP and NELP status. In our ED, providers typically review the triage note prior to assigning themselves to new patients. We found that TTP delays were higher in patients with a NELP with a triage note identifying their NELP status. Differences in TTP associated with triage note-unidentified patients with a NELP compared with triage-identified patients with a NELP raise questions regarding perceived barriers among providers to using interpreter services as well as elements of subconscious bias. This concern for bias is likely reflected in the minimal difference in TTP among patients with an ESI of 2 for whom providers have a greater sense of urgency, diminishing the ability to overlook or “skip” these patients, regardless of their language preference. We specifically selected TTP as an outcome that was insulated from the effect of delays in interpreter services because the request for interpreter is made after provider assignment. However, other operational delays unrelated to provider bias such as delays in communication during the rooming process, transport, or brief interactions at the bedside with other members of the care team may occur that increase TTP. Finally, the causal effect of a triage note identifying NELP status remains unknown because we did not determine if the provider actually read or incorporated knowledge of the text in their evaluation.

Additionally, prolonged TTP for patients with a NELP during times of stress in the ED highlights a differential impact of crowding on marginalized populations, a concern raised by previous studies.^16^^,^^21^ The findings of our study are consistent with those of a 2021 study assessing care metrics for patients with a NELP, which showed that patients with a NELP spend more time in the ED before being transported to the intensive care unit and experience higher mortality rates compared to patients with an ELP.^22^ As boarding and WR volume increases, assessments of how marginalized populations may be uniquely impacted must be incorporated.

Although our study was performed at a single site, unique knowledge of our site allowed identification of workflow factors influenced by crowding and high system stress within the ED. We believe this facilitated more accurate identification of differences in TTP between triage-identified patients with a NELP and an ELP. Additionally, this site-specific analysis allows for tailored interventions, such as the possibility of assigning a provider to a patient once an interpreter has been requested at triage, changes to availability of interpreter services in response to ED crowding, or other quality improvement interventions. Other sites undertaking similar studies should design site-specific interventions that are tailored to their workflow. As ED crowding increases, replication of this study in centers with significant NELP patient populations is important to ensure equitable care and to further understand how regional non-English language prevalence, in both providers and patients, affects TTP. With the percentage of patients with a NELP on the rise,^23^ increasing our knowledge of the logistics involved in caring for these patients is critical in establishing necessary guidelines on best practices within the field of emergency medicine.

## Author Contributions

AR and CF conceived the study and designed the study. CC and CF extracted data from the electronic health record. tAS, EL, MP, and DH completed review of triage notes. CF analyzed the data and oversaw and managed all aspects of data collection and cleaning. AR drafted the manuscript, and all authors contributed substantially to its revision. AR and CF take responsibility for the paper.

## Funding and Support

By *JACEP Open* policy, all authors are required to disclose any and all commercial, financial, and other relationships in any way related to the subject of this article as per ICMJE conflict of interest guidelines (see www.icmje.org). The authors have stated that no such relationships exist.

## Conflict of Interest

All authors have affirmed they have no conflicts of interest to declare.
