# Supplementary material for: Increased Time to Provider for Patients With a Non-English Language Preference: A Retrospective Cohort Study
Source: J Am Coll Emerg Physicians Open. 2025 Aug 29;6(5):100239. doi: 10.1016/j.acepjo.2025.100239 (PMC12414885; doi:10.1016/j.acepjo.2025.100239)
Supplement: Supplementary Figures 1-3 and Supplementary Table 1 [file mmc1.pdf]

Figure S1. Emergency Department Workflow

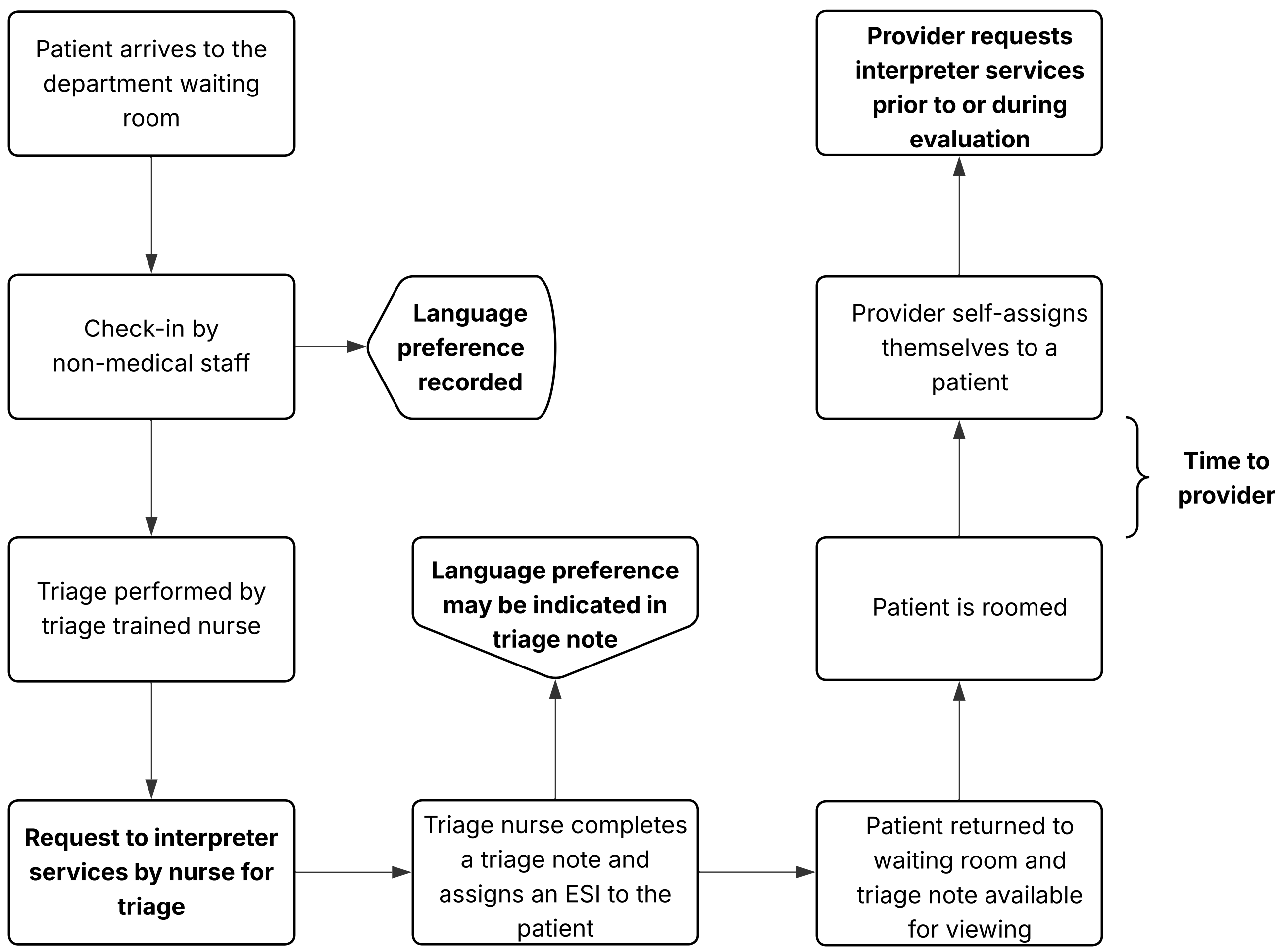

Figure S1. Emergency Department Workflow. Patients are asked their language preference as part of the intake process on their arrival to the department. Each patient is then called from the waiting room into a triage room, where they undergo evaluation by an emergency department nurse. Based on this evaluation, the nurses assigns an emergency severity index (ESI) to the patient and documents a triage note for the patient. The triage nurse will utilize an in-person or over-the-phone interpreter as needed. The patient then returns to the waiting room until a treatment space is available. Upon rooming, the time to provider measurement starts and stops when a provider self-assigns themselves to the patient. Typically, the provider requests interpreter services after assignment or during evaluation of the patient.

Triage Note Labeling

Record # [record_id]

Who are you?

*There may be a default assignment pre-checked.

- ◯ Anne
- ◯ Asmaa
- ◯ Chris
- ◯ Morgan
- ◯ Erica
- ◯ David

This section collections information about the triage note and its communcation to clinicians regarding the patient's preferred language.

Triage Note:

[triage_note]

Does this triage note contain text that identifies the patient as having a non-English preferred language?

*Mark No if the triage note is blank.

- ◯ Yes
- ◯ No

Does this triage note have text that indicates a family member, friend or other visitor is interpreting or translating for the patient?

*If the visitor is present but NOT interpreting/translating then mark no.

*If the note does not explicitly say that the visitor is translating then mark no.

- ◯ Yes
- ◯ No

Does the text of the triage note indicate that the patient as declined interpreter services?

- ◯ Yes
- ◯ No

According to the triage note, what is the patient's preferred language?

*Select "No specific language" when you know that the patient has NELP but the preferred language is not mentioned in the note.

- ◯ Spanish
- ◯ Arabic
- ◯ Mandarin
- ◯ Other (free text)
- ◯ No specific language

What other language?

____

Figure S2

**[triage_note]**	
Please select what other domains of biasing or stigmatizing text are present in this triage note?<br><br>*Mark No stigmatizing language if the triage note is blank.	☐ Comments on patient behavior<br>☐ Discounting of symptoms<br>☐ Medical stigma<br>☐ Negative patient descriptors<br>☐ SES<br>☐ SUD<br>☐ Weight related stigma<br>☐ Race or ethnicity<br>☐ Safety concern<br>☐ Other<br>☐ No stigmatizing language present<br>(Check all that apply)
What other biasing or stigmatizing text is present?	______________________________
Survey URL	______________________________

Figure S3

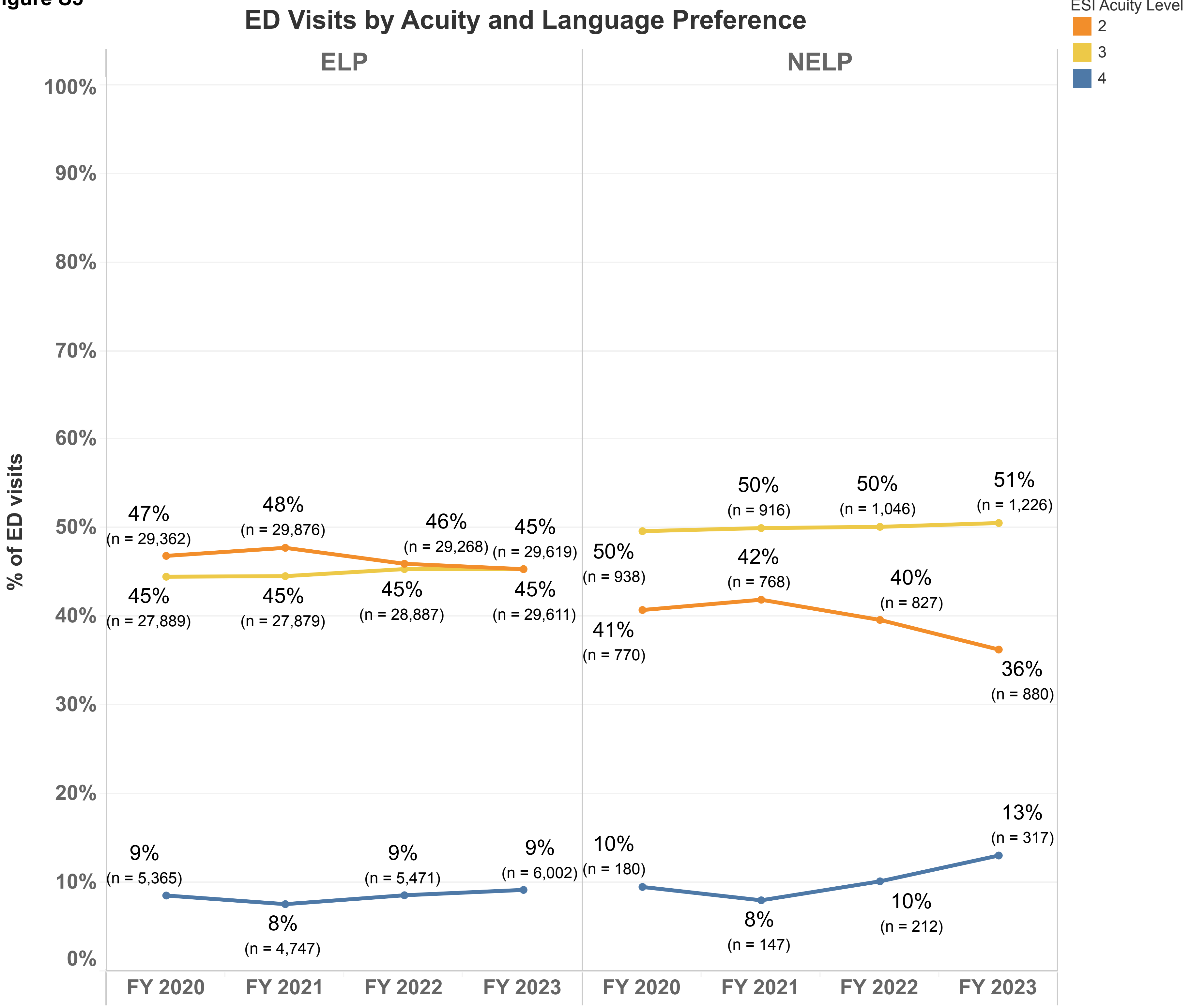
ED Visits by Acuity and Language Preference
ESI Acuity Level
2
3
4
ELP
NELP
% of ED visits
100%
90%
80%
70%
60%
50%
40%
30%
20%
10%
0%
47%
(n = 29,362)
48%
(n = 29,876)
46%
(n = 29,268)
45%
(n = 29,619)
45%
(n = 27,889)
45%
(n = 27,879)
45%
(n = 28,887)
45%
(n = 29,611)
9%
(n = 5,365)
8%
(n = 4,747)
9%
(n = 5,471)
9%
(n = 6,002)
50%
(n = 938)
50%
(n = 916)
50%
(n = 1,046)
51%
(n = 1,226)
41%
(n = 770)
42%
(n = 768)
40%
(n = 827)
36%
(n = 880)
10%
(n = 180)
8%
(n = 147)
10%
(n = 212)
13%
(n = 317)
FY 2020
FY 2021
FY 2022
FY 2023
FY 2020
FY 2021
FY 2022
FY 2023

Table S1. NELP Observations by Language

Language (Top 10)	Triage Note Identified	Triage Note Unidentified	Grand Total
SPANISH	1,182 (35.0%)	1,464 (30.2%)	2,646 (32.2%)
ARABIC	860 (25.5%)	1,340 (27.6%)	2,200 (26.7%)
CHINESE, MANDARIN	393 (11.6%)	620 (12.8%)	1,013 (12.3%)
KOREAN	84 (2.5%)	143 (2.9%)	227 (2.8%)
FRENCH	47 (1.4%)	169 (3.5%)	216 (2.6%)
RUSSIAN	108 (3.2%)	95 (2.0%)	203 (2.5%)
ALBANIAN	94 (2.8%)	94 (1.9%)	188 (2.3%)
JAPANESE	55 (1.6%)	130 (2.7%)	185 (2.2%)
URDU	53 (1.6%)	103 (2.1%)	156 (1.9%)
VIETNAMESE	41 (1.2%)	42 (0.9%)	83 (1.0%)
Other	458 (13.6%)	652 (13.4%)	1,110 (13.5%)
Grand Total	3,375 (100.0%)	4,852 (100.0%)	8,227 (100.0%)
